# Non-islet cell tumor hypoglycemia caused by intrathoracic solitary fibrous tumor: a case report

**DOI:** 10.1186/s13019-016-0463-6

**Published:** 2016-04-09

**Authors:** Masahiro Kitada, Shunsuke Yasuda, Nana Takahashi, Satoshi Okazaki, Kei Ishibashi, Satoshi Hayashi, Yoshinobu Ohsaki, Naoyuki Miyokawa

**Affiliations:** Department of Respiratory Center, Asahikawa Medical University, Midorigaoka-Higashi 2-1-1-1, Asahikawa, Hokkaido 078-8510 Japan; Department of Clinical Pathology, Asahikawa Medical University, Midorigaoka-Higashi 2-1-1-1, Asahikawa, Hokkaido Japan

**Keywords:** Solitary fibrous tumor, Non-islet cell tumor hypoglycemia, Hypoglycemia

## Abstract

**Background:**

Non-islet cell tumor hypoglycemia (NICTH) is defined as a form of hypoglycemia caused by an extrapancreatic tumor. Solitary fibrous tumor (SFT) associated with hypoglycemia is rare.

**Case presentation:**

A 76-year-old woman, who had frequently experienced hypoglycemic symptoms such as presyncope for the prior 6 months, visited our hospital to undergo detailed examinations. Her fasting glucose level was low at 49 mg/dl. The blood levels of IRI and C-peptide were also low at 0.2 μU/ml and 0.21 ng/ml, respectively. Chest computed tomography revealed a mass measuring 15 cm in the left thoracic cavity. Percutaneous needle biopsy yielded a diagnosis of intrathoracic SFT associated with NICTH. The tumor was removed by video-assisted thoracoscopic surgery. Histological examination showed a tumor composed of simple spindle-shaped cells with an irregular arrangement. Immunohistochemical staining was positive for CD34, bcl-2, and vimentin and negative for alpha SMA and mesothelin. These results confirmed the diagnosis of SFT. Her hypoglycemic symptoms resolved rapidly after surgery. The clinical course has since remained favorable with no signs of recurrence.

**Conclusion:**

We report a case of non-islet cell tumor hypoglycemia caused by intrathoracic SFT. The high-molecular-weight IGF-II produced by the tumor has been regarded as the cause of NICTH.

## Background

Non-islet cell tumor hypoglycemia (NICTH) is defined as hypoglycemia caused by an extrapancreatic tumor. NICTH can occur in patients with mesenchymal tumors, hepatocellular carcinomas, and other extrapancreatic tumors. Solitary fibrous tumor (SFT) associated with hypoglycemia is rare at approximately 4 % [1, 2]. The high-molecular-weight IGF-II produced by the tumor has been regarded as the cause of NICTH [3, 4]. Herein, we report a case with a surgically treated intrathoracic SFT, diagnosed based on recurrent episodes of hypoglycemia.

## Case presentation

**Fig. 1:**
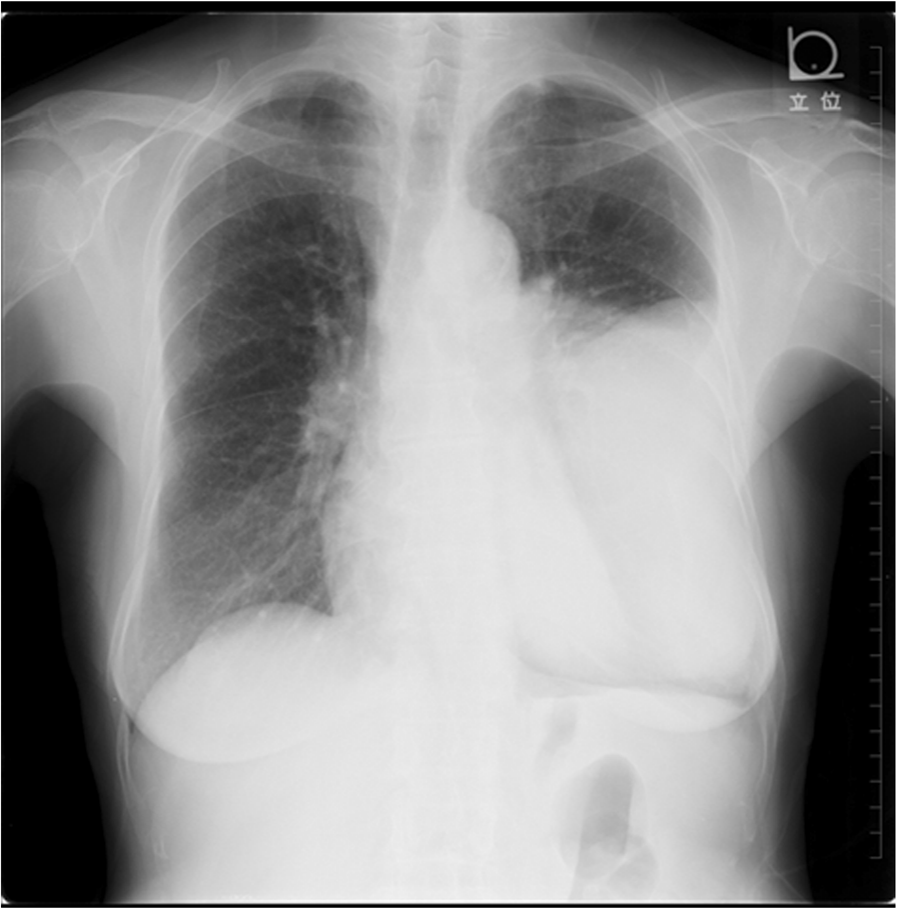
Chest radiography shows a huge mass in the left thoracic cavity

**Fig. 2:**
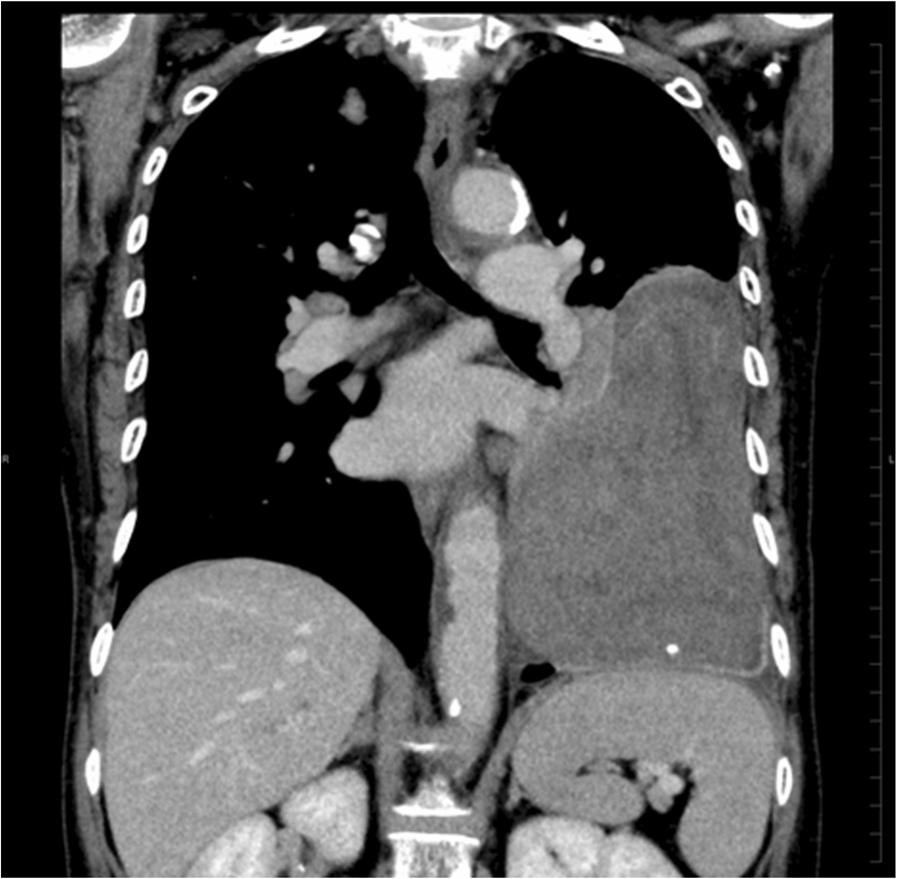
Chest computed tomography reveals a mass measuring 15 cm with no invasion to the thoracic wall

**Fig. 3:**
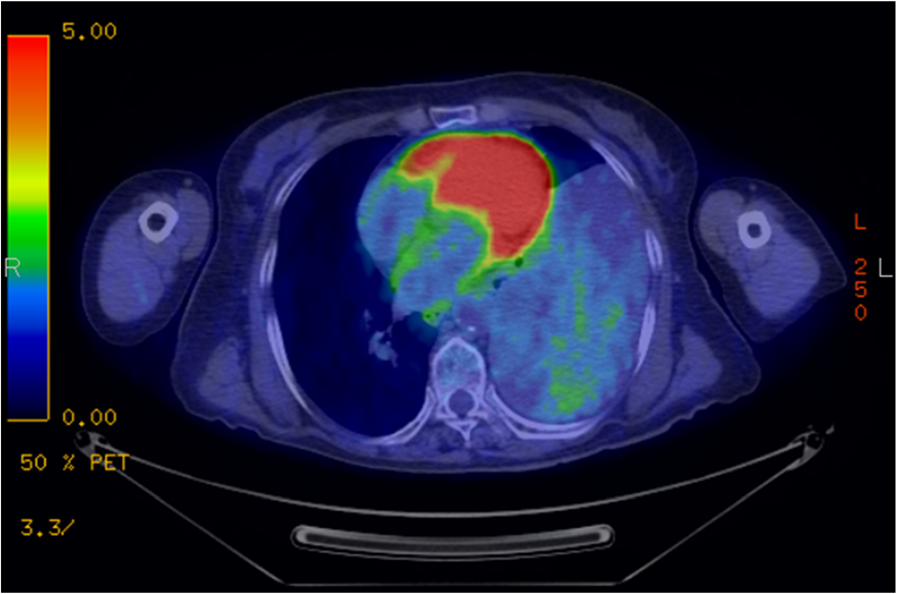
FDG-positron emission tomography shows low FDG uptake in the intrathoracic mass (a low SUV)

**Fig. 4:**
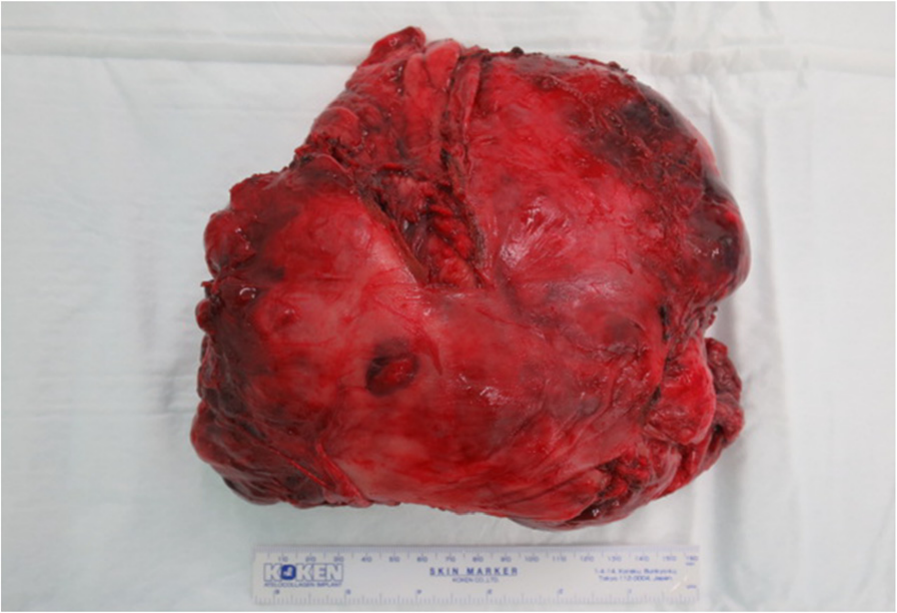
The excised specimen was a lobulated mass with dimensions of 16 × 15 × 8 cm

**Fig. 5:**
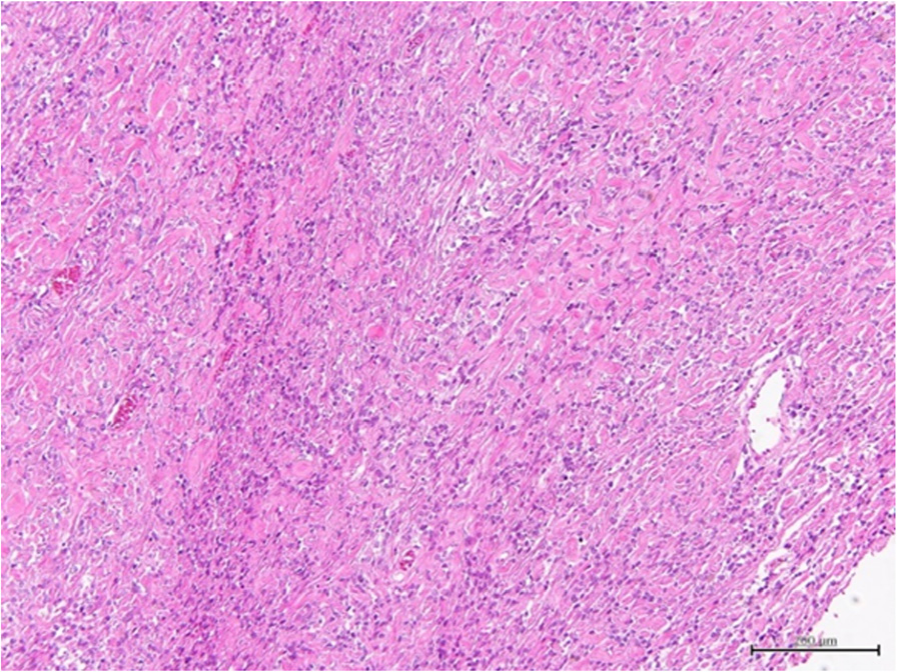
Histological examination (HE × 100) showed a tumor composed of simple spindle-shaped cells arranged irregularly without features suggestive of malignancy

**Fig. 6:**
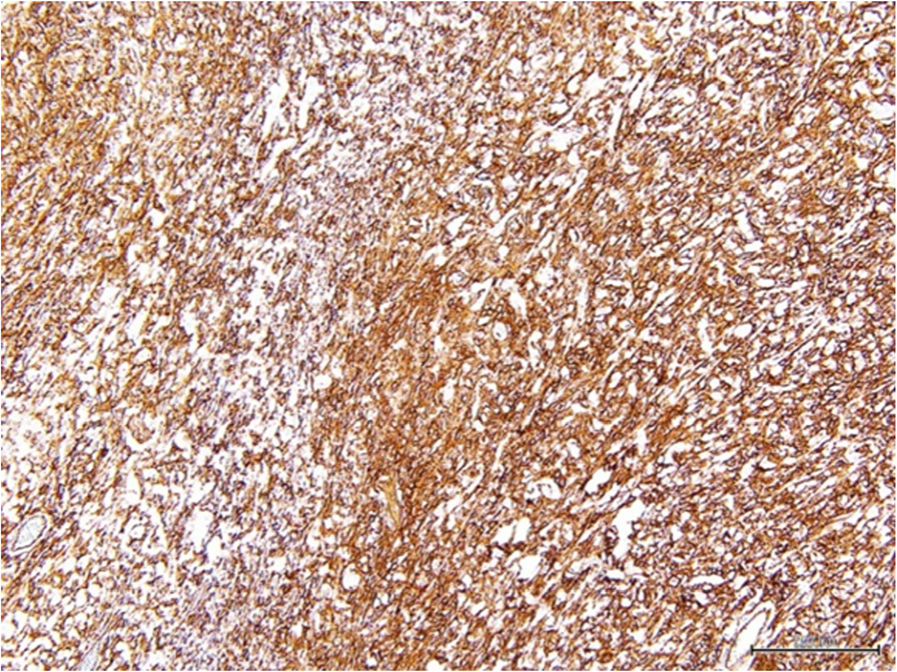
Immunohistochemical staining (CD34 × 100) was positive for CD34 and bcl-2

A 76-year-old woman, who had frequently experienced hypoglycemic symptoms such as presyncope for the prior 6 months, visited the internal medicine department of our hospital. Family history and past medical history were unremarkable. Physical examination showed a height of 155 cm, weight of 52 kg, blood pressure of 145/68 mmHg, and heart rate of 90 beats/min. There were no remarkable findings except for diminished breath sounds in the left middle and lower lobes of the lung. Her fasting glucose level was low, at 49 mg/dl. Her blood levels of IRI and C-peptide were also low at 0.2 μU/ml and 0.21 ng/ml, respectively. Chest radiography showed a huge mass in the left thoracic cavity (Fig. 1). Chest computed tomography revealed a mass measuring 15 cm with no invasion to the chest wall (Fig. 2). Positron emission tomography showed low FDG uptake in the intrathoracic mass (a low SUV) (Fig. 3). There were no lesions in other organs. Percutaneous needle biopsy was performed and SFT was thus diagnosed. The patient was diagnosed with intrathoracic SFT associated with NICTH and surgical treatment was planned. The tumor was removed by video-assisted thoracoscopic surgery. The excised specimen was a lobulated mass measuring 16 × 15 × 8 cm (Fig. 4). Histological examination showed a tumor composed of simple spindle-shaped cells with an irregular arrangement (Fig. 5). Immunohistochemical staining was positive for CD34 (Fig. 6), bcl-2, and vimentin, and negative for alpha SMA, mesothelin, s-100. The diagnosis of SFT was thus confirmed. Her hypoglycemic symptoms resolved rapidly after surgery. The clinical course was favorable with no signs of recurrence.

## Discussion

It is known that NICTH can occur in patients with mesenchymal-derived tumors, hepatocellular carcinomas, and other extrapancreatic tumors. Approximately 4 % of SFTs are reportedly associated with hypoglycemia [1–4]. NICTH is defined as hypoglycemia caused by a non-beta islet cell tumor. This condition is often associated with excessive production of high-molecular-weight IGF-II from the tumor and suppressed levels of serum albumin and IGF-I [5, 6]. NICTH is diagnosed by detecting the presence of an extrapancreatic tumor and by excluding other diseases that can cause hypoglycemia, such as liver failure, adrenal failure, and insulinoma. Although testing of high-molecular-weight IGF-II from the tumor by the Western immunoblot method or gel-filtration is more accurate for establishing the diagnosis, these procedures often are not feasible in many routine clinical settings. Therefore, the diagnosis is frequently made based on the clinical course. In the present case, although the production of high-molecular-weight IGF-II from the tumor was not demonstrated, we considered her hypoglycemia to be attributable to NICTH because the patient had no other diseases that could have caused hypoglycemia (such as the aforementioned liver failure, adrenal failure, and insulinoma) and levels of IRI and C-peptide were low. Furthermore, her hypoglycemic symptoms resolved rapidly after resection of the tumor.

Insulin-like growth factors (IGFs) consist of IGF-I and IGF-II: IGD-I has important growth-promoting features while IGF-II has an insulin-like hypoglycemic effect. The normal-molecular-weight form of IGF-II is bound to IGF-binding protein, forming a trimer. On the other hand, in NICTH, high-molecular-weight IGF-II produced by the tumor forms a dimer which tends to result in extravascular leakage and tissue penetration, leading to hypoglycemia [7].

Resection of the primary tumor is the first-line treatment for NICTH [8]. When curative resection is difficult, chemotherapy aimed at downsizing the tumor is a potential treatment option. There are also reports describing treatment with steroids or growth hormone as being effective [9, 10]. Steroids are known to have an effect not only on glycogen degradation and gluconeogenesis but also on the inhibition of IGF-II production, although the underlying mechanism has yet to be elucidated [11]. Further investigations are required to clarify the mechanisms underlying the development of NICTH.

## Conclusion

We report a case of non-islet cell tumor hypoglycemia caused by intrathoracic SFT. The high-molecular-weight IGF-II produced by the tumor has been regarded as the cause of NICTH.

## Consent

Written informed consent was obtained from the patients for publication of this case report and any accompanying images. A copy of the written consent is available for review by the Editor-in Chief of this journal.
